# Surveillance or metastasis-directed Therapy for OligoMetastatic Prostate cancer recurrence (STOMP): study protocol for a randomized phase II trial

**DOI:** 10.1186/1471-2407-14-671

**Published:** 2014-09-15

**Authors:** Karel Decaestecker, Gert De Meerleer, Filip Ameye, Valerie Fonteyne, Bieke Lambert, Steven Joniau, Louke Delrue, Ignace Billiet, Wim Duthoy, Sarah Junius, Wouter Huysse, Nicolaas Lumen, Piet Ost

**Affiliations:** Department of Urology, Ghent University Hospital, Ghent, Belgium; Department of Radiotherapy, Ghent University Hospital, Ghent, Belgium; Department of Urology, AZ Maria Middelares Gent, Ghent, Belgium; Department of Nuclear Medicine, Ghent University Hospital, Ghent, Belgium; Department of Urology, University Hospitals Leuven, Leuven, Belgium; Department of Radiology, Ghent University Hospital, Ghent, Belgium; Department of Urology, AZ Groeninghe, Kortrijk, Belgium; Department of Radiotherapy, AZ Sint-Lucas Gent, Ghent, Belgium; Department of Radiotherapy, CH Mouscron, Mouscron, Belgium

**Keywords:** Oligometastases, Prostate cancer, Salvage treatment, Stereotactic body radiotherapy, Salvage lymph node dissection, Active surveillance, Androgen deprivation therapy, Quality of life, Survival

## Abstract

**Background:**

Metastases-directed therapy (MDT) with surgery or stereotactic body radiotherapy (SBRT) is emerging as a new treatment option for prostate cancer (PCa) patients with a limited number of metastases (≤3) at recurrence – so called “oligometastases”. One of the goals of this approach is to delay the start of palliative androgen deprivation therapy (ADT), with its negative impact on quality of life. However, the lack of a control group, selection bias and the use of adjuvant androgen deprivation therapy prevent strong conclusions from published studies.

The aim of this multicenter randomized phase II trial is to assess the impact of MTD on the start of palliative ADT compared to patients undergoing active surveillance.

**Methods/Design:**

Patients with an oligometastatic recurrence, diagnosed on choline PET/CT after local treatment with curative intent, will be randomised in a 1:1 ratio between arm A: active surveillance only and arm B: MTD followed by active surveillance. Patients will be stratified according to the location of metastasis (node vs. bone metastases) and PSA doubling time (≤3 vs. > 3 months). Both surgery and SBRT are allowed as MDT. Active surveillance means 3-monthly PSA testing and re-imaging at PSA progression. The primary endpoint is ADT-free survival. ADT will be started in both arms at time of polymetastatic disease (>3 metastatic lesions), local progression or symptoms. The secondary endpoints include progression-free survival, quality of life, toxicity and prostate-cancer specific survival.

**Discussion:**

This is the first randomized phase 2 trial assessing the possibility of deferring palliative ADT with MDT in oligometastatic PCa recurrence.

**Trial registration:**

Clinicaltrials.gov identifier: NCT01558427

## Background

Prostate cancer (PCa) is the most frequent tumour in males and PCa death is attributed to metastatic disease in the majority of patients [1]. The first line treatment of metastatic PCa is lifelong androgen deprivation therapy (ADT) by means of surgical or medical castration [1]. Although this strategy delays PCa progression, it is associated with numerous side effects impacting quality-of-life and general health [2, 3]. There is no proven overall survival benefit of immediate ADT over deferred ADT in metastatic patients [1]. It is therefore an option in the EAU guidelines to suggest an active surveillance protocol for well-informed asymptomatic patients with PCA metastases [1].

In analogy with other solid tumours, eradication of these oligometastases by means of metastases directed therapy (MDT) with stereotactic body radiotherapy (SBRT) or surgery is a promising and emerging way to delay disease progression and postpone systemic treatment without major treatment toxicity [4]. However, two major difficulties complicate assessment of the benefit of radical treatment for oligometastastatic prostate cancer recurrence.

Firstly, the identification of patients with oligometastatic disease is challenging. The traditional imaging studies such as bone scan and computed tomography lack sufficient sensitivity to detect low volume metastatic disease at low PSA levels [5]. Consequently, these imaging modalities are not recommended to screen patients for metastases until the PSA reaches 10 ng/ml^19^. Choline positron emitting tomography (PET) with computed tomography (CT) might be a good candidate for the identification of low volume metastases with a pooled sensitivity and specificity exceeding 85% on a per-patient basis in the recurrent setting [6, 7].

Secondly, the apparent achievement of MDT for oligometastatic PCa in postponing palliative ADT and in slowing down progression has only been observed in single arm studies [8, 9]. The true benefit of this MDT can only be assessed through randomization versus a control group. Otherwise, it could be that the promising results are only due to selection of fit patients with very slow-growing tumors, rather than the result of treatment intervention itself.

The current study will address these shortcomings by randomizing MDT versus active surveillance. The active surveillance arm will inform us about the natural progression of oligometastatic PCa.

This study is the first randomized study in this setting and will employ a randomized phase II design to determine which arm is justified to be tested in a subsequent phase III trial.

## Methods/design

This study is approved by the Ethics committee of the Ghent University Hospital (EC2012/156) and is registered on clinicaltrials.gov (NCT01558427). The protocol is designed according to the clinical-state model [10]: rising PSA and clinical metastases state in non-castrate patients. Patients diagnosed with oligometastases (up to 3 N1 or M1a/b lesions [1]) will be entered in a randomized Phase II trial. In the experimental arm A, patients will undergo active clinical surveillance with the start of ADT at progression. In arm B, patients will receive SBRT or surgery of the oligometastases, followed by active clinical surveillance.

### Objectives

- Primary endpoint
  - ○ Androgen deprivation therapy free survival ADT will be started in both arms at time of polymetastatic disease, local progression (defined below) or symptoms. In case of a metachronous oligometastatic recurrence in arm A, a retreatment with radiotherapy or surgery is allowed. Calculation will start from randomization until ADT is started.
- Secondary endpoints
  - ○ Quality of life scoring using the EORTC QLQ-C30 supplemented with QLQ-PR25. Raw scores will be transformed to a linear scale ranging from 0 to 100. The results will be presented in accordance with recent guidelines for reporting HRQOL RCTs [11].
  - ○ Assessment of quality-adjusted-life-years with the EuroQol classification system (EQ-5D) [12]. A written consent to use this system has been obtained from the EuroQol Group Foundation.
  - ○ Acute and late toxicity due to radiotherapy will be scored using the Common toxicity criteria version 4.0 [13]. Surgical complications will be scored using Clavien-Dindo Classification [14]. Other surgical related morbidity (intra-operative complications (blood loss, injury to pelvic or intra-abdominal organs…) and duration of hospital stay) will also be recorded.
  - ○ Time to castration-resistant disease is calculated from randomization until development of castration-resistant disease as defined by the EAU guidelines [1].
  - ○ Progression-free survival:
    - ▪ 3 types of progression are defined and definitions of progression are used and registered according to the recommendations of the prostate cancer clinical trials working group [10]. Calculation will start from randomization until progression or death.
    - ▪ PSA or biochemical progression:
      - In case of decline from baseline: record time from randomization to first PSA increase that is ≥25% and ≥ 2 ng/ml above the nadir OR that is ≥25% and rises above the pre-treatment PSA value and which is confirmed by a second value 3 or more weeks later.
      - In case of no decline from baseline: PSA increase that is ≥25% and ≥ 2 ng/ml after 3 months if baseline PSA is ≥2 ng/ml. PSA increase that is ≥25% after 3 months if baseline PSA is < 2 ng/ml.
      - Local progression for soft-tissue and bone lesions: Each metastasis is a target lesion independently assessed for response with the RECIST criteria [15]. In addition, metastases (particularly osseous) with a metabolic complete response on bone or PET scan are scored as complete response in the absence of progression on CT scan.
    - ▪ Distant progression: appearance of new metastatic lesions.
      - Prostate cancer specific survival will be calculated from randomization until PCa death. Overall survival will be calculated from randomization until death from any cause.
      - Time to first symptomatic event will be calculated from randomization until the event of symptoms due to metastatic disease.

### Inclusion criteria

- Histologically proven diagnosis of PCa
- PCa patients with a biochemical recurrence following treatment with curative intent (radical prostatectomy, primary radiotherapy or a combination of both) as defined by the EAU guidelines [1].
- A maximum of 3 extracranial metastases in any organ system diagnosed on choline PET-CT.
- Controlled primary tumor
  - ○ Patients in the postoperative setting should have received postoperative radiotherapy to the prostate bed. In case the PSA > 2 ng/ml in the postoperative setting patients are eligible if a multiparametric MRI of the prostate bed rules out a local relapse or a negative biopsy of the prostate bed is performed^37^. Patients after primary radiotherapy should undergo MRI of the prostate according to the European Society of Urogenital Radiology (ESUR) guidelines to rule out local relapse [16]. In case of a suspicious lesion, a biopsy should confirm local recurrence and patients should be referred for local salvage prostatectomy when distant metastases are ruled out. If MRI rules out local relapse, patients are eligible.
- WHO performance state 0-1
- Age ≥18 years old
- Willing to provide a signed informed consent
- Patient presented at the multidisciplinary tumour board of the local hospital in which the therapy will be given.

### Exclusion criteria

- Serum testosterone level <50 ng/ml
- Symptomatic metastases
- Patients with oligometastases that have been previously treated.
- PSA rise while on active treatment (LHRH-agonist, LHRH-antagonist, anti-androgen, maximal androgen blockade, oestrogen)
- Previous treatment with a cytotoxic agent for PCa
- Treatment during the past month with products known to influence PSA levels (e.g. fluconazole, finasteride, corticosteroids,…)
- Disorder precluding understanding of trial information or informed consent

### Evaluation and randomization

Patients must be restaged within 4 weeks prior to randomization with 18F or 11C-choline PET-CT. MRI full spine and bony pelvis or MRI whole body is optional, but recommended in case of equivocal findings. Bone scintigraphy is not required.

The study will employ a 1:1 randomization between arm A: arm B. Patients will be stratified according to PSA doubling time and (≤3 vs. > 3 months) and initial localization of metastases (node vs bone or visceral).

### Interventions

1. Arm A: Active clinical surveillance
  Defined as 3-monthly clinical examination and serum PSA measurement. Restaging will be performed in case of symptomatic progression or PSA progression:
    ▪ A PSA increase that is ≥25% and ≥ 2 ng/ml if baseline PSA is ≥2 ng/ml.
    ▪ A PSA increase that is ≥25% if baseline PSA is < 2 ng/ml.
  ADT will be started at time of polymetastatic disease, local progression (defined above) or symptoms. The type of ADT is left to the discretion of the treating physician. Both anti-androgen monotherapy, LHRH agonist or antagonists and maximal androgen deprivation therapy are allowed. Both intermittent and continuous ADT are allowed.
2. Arm B
  - SBRT and surgery.
    All patients randomized into arm A will be presented at the multidisciplinary urology tumour board prior to treatment. The choice between SBRT or surgery will depend on localization and size of the metastases, the nearby organs-at-risk and previous treatments in the vicinity of the metastases. After reaching a consensus, the patient will be informed about the options for treatment.
  - SBRT target and organ-at-risk definition.
    All patients will receive a CT simulation in supine position with 2 mm CT slice thickness through the tumour site. The planning simulation should cover the target and all organs at risk. A typical scan length should extend at least 10 cm superior and inferior beyond the treatment field borders. Support devices to increase patient comfort will be chosen depending on the tumour localisation. The isocenter will be determined on the CT-simulator with marking of laser lines on the patient. Imaging data will be transferred to the treatment planning system.
    For all lesions, the Gross Target Volume (GTV) will be defined as all visible tumor by combining iconographic and metabolic information. No additional margin will be added for microscopic spread of disease. The GTV will be expanded with 2-5 mm to the Planning Target Volume (PTV) to account for organ motion and setup error. Margins depend on the site irradiated with 2 mm margins for bony lesions, 3 mm for nodes and 5 mm for other sites. The type of organ at risk delineated depends on the localization of the metastasis. A Planning Organ at Risk Volume (PRV) expansion of 2-5 mm will be added for OAR such as the spinal cord, oesophagus, intestine,… (if applicable), and dose constraints apply to this PRV. It is strongly recommended that dose constraints not be exceeded. If a dose constraint cannot be achieved due to overlap of the target with an organ at risk or its PRV, the total dose can be lowered in order to meet the constraint.
    For spinal lesions, a pre-treatment axial MRI is required to assess the extent of disease and position of the cord. This must be fused with the planning CT scan.
  - Radiotherapy treatment planning and dose prescription:
    IMRT (static or rotational) treatment planning will be dependent the localization of the metastasis. Dose constraints for organ at risks will be in accordance with the recommendations from the report of the AAPM task group 101 [17]. A total dose of 30 Gy (80% of the maximal dose) will be delivered in 3 fractions and fractions will be separated >48 h and <96 h [18, 19]. Treatment will be prescribed to the periphery of the target (80% of the dose (=30 Gy), should cover 90% of the PTV). In case of violation of dose constraints to the organs at risk, the prescription will be adapted accordingly. If multiple targets will be irradiated and the targets are more than 10 cm apart in the cranio-caudal direction, multiple isocenters are needed with a CBCT prior to every treatment for every isocenter. Patient immobilization devices can be used according to the institutional policy.
  - Radiotherapy delivery and verification:
    In order to ensure patient safety and effective treatment delivery the following measurements are taken:
    Prior to treatment, all plans are discussed and approved at the daily radiotherapy rounds after prior verification by the treating physician.
    All dose delivery for intensity-modulated plans will be confirmed before treatment by physics staff.
    At each fraction, a cone-beam CT (CBCT) will be used for patients’ set-up and target verification prior to treatment. In case of multiple isocenters, every isocenter will be verified separately with CBCT.
    Quality assurance: all plans will be verified using the Delta(4) diode array phantom prior to treatment delivery [20].
  - Surgical procedures:
    The surgical technique to be used is at the discretion and expertise of the surgeon but must be in accordance to the best surgical practice available. A minimally invasive technique is preferred, but not obligatory. For pelvic nodal metastases, in case of a previous extended pelvic lymph node dissection (ePLND) or pelvic radiotherapy, only the suspicious lymph node will be removed. If no ePLND has been performed previously, a salvage ePLND will be preferred. In case of retroperitoneal nodes, only the suspicious node will be removed.
  - Androgen deprivation therapy:
    ADT will be started as in arm A. In case of a metachronous oligometastatic recurrence, a retreatment with SBRT or surgery is allowed.

### Follow-up

Patients will be seen every 3 months post-randomization (Table 1). At each visit, a history and physical examination will conducted with recording of the toxicity. The QOL questionnaires will be concluded at each visit. Choline PET-CT will be repeated at PSA or symptomatic progression. Additional Imaging or laboratory investigations should be carried out at the discretion of the treating physician, based on findings in the history or physical, and additional treatment (e.g. ADT).

**Table 1:** **Study visits and procedures**

	At inclusion	3-monthly	At PSA or symptomatic progression
Eligibility check	*		
Informed consent	*		
PET-CT	*		*
QOL questionnaire	*	*	*
Toxicity assessment	*	*	*
PSA measurement	*	*	*
History and physical examination	*	*	*

*reflects the timepoint of a certain assessment.

### Statistical analysis

#### Sample size

This study will employ a randomized phase II design to determine which arm is justified to be tested in a subsequent phase III trial. The study will therefore be designed with an alpha and beta = 0.20 (as recommended for phase II randomized trials [21, 22]). It is estimated that the median delay to start palliative ADT after metastasis-directed therapy is approximately 24 months [4]. There will be a 1:1 randomization between Arm 1 and Arm 2. Patients will be stratified according to PSA doubling time (≤3 vs. > 3 months) and initial localization of metastases (node vs bone or visceral). In order to detect a 12-month difference in the studied endpoint from 12 to 24 months, a total of 58 patients will be needed. Assuming a 5% rate of loss to follow-up, a total of 62 patients will be accrued over 36 months with 12 months of additional follow-up. We expect an accrual rate of 20 patients per year.

#### Data analysis

Patients will be analysed in the groups to which they are assigned (intention-to-treat). The primary endpoint androgen deprivation therapy-free survival will be calculated using Kaplan-Meier actuarial analyses. Pre-planned subgroup analysis will occur based on stratification variables using the log-rank test. Survival times are defined from the day of randomization until an event (start of ADT) or last follow-up. Cases will be censored at last follow up visit if no ADT was started. Multivariate analysis will be performed according to the Cox-Regression method.

Overall survival and progression-free survival (local, biochemical and clinical progression free survival) will be evaluated in the same way as the primary endpoint. All p values are set at 0.05. Statistical analysis will be performed with SPSS (IBM Corp, Somers, NY, USA).

## Discussion

The standard treatment options for PCa patients diagnosed with metastatic progression following primary treatment have remained unchanged over the past years [23], with ADT being the cornerstone of treatment [1]. Several small, single arm observational studies have been reported with promising results [4, 8, 9, 19, 24–26]. However the rather random use of a multimodality approach with adjuvant ADT and prophylactic nodal irradiation in these studies makes it difficult to make any definite conclusions. This is the first randomized phase 2 trial that will asses the possibility of deferring palliative ADT and cancer progression with MDT by means of SBRT or surgery. The inclusion of an active surveillance arm will improve our insights in the natural progression of oligometastatic PCa.

## Abbreviations

PCa: Prostate cancer
ADT: Androgen deprivation therapy
EAU: European Association of Urology
PET-CT: Positron emission tomography
CT: Computed tomography
MRI: Magnetic resonance imaging
AS-MRI: Axial skeleton MRI
WB-MRI: Whole body MRI
PSA: Prostate specific antigen
SBRT: Stereotactic body radiotherapy
ePLND: Extended pelvic lymph node dissection
IMRT: Intensity modulated radiotherapy
AAPM: American Association of Physicists in Medicine
GTV: Gross target volume
PTV: Planned target volume
PRV: Planning organs at risk volume
RT: Radiotherapy
QOL: Quality of life
EORTC: European Organization for Research and Treatment of Cancer
RTOG: Radiation Therapy Oncology Group
WHO: World Health Organization
ESUR: European Society of Urogenital Radiology
LHRH: Luteinizing hormone releasing hormone.
